# The Critical Role of Adenylate Kinase in Regulating the Glycolysis Rate in Cells

**DOI:** 10.3390/ijms27052479

**Published:** 2026-03-08

**Authors:** Michael V. Martinov, Fazoil I. Ataullakhanov, Victor M. Vitvitsky

**Affiliations:** Center for Theoretical Problems of Physico-Chemical Pharmacology, Russian Academy of Sciences, Moscow 109029, Russia; martinov.michael@gmail.com (M.V.M.); ataullakhanov.fazly@gmail.com (F.I.A.)

**Keywords:** adenylate kinase, erythrocyte, glycolysis, mathematical modeling, muscle

## Abstract

The role of adenylate kinase in regulating the glycolysis rate and the potential contribution of the adenylate kinase reaction to ATP production were examined using mathematical models of energy metabolism in human erythrocytes and resting anaerobic mammalian skeletal muscle. The adenylate kinase reaction was shown to play a critical role in the regulation of cellular energy metabolism. Through the action of adenylate kinase, small changes in intracellular [ATP] give rise to large changes in [AMP], a potent activator of glycolytic flux via the activation of phosphofructokinase (PFK). This mechanism ensures an increase in the glycolytic rate as [ATP] decreases within the physiological range of ATP concentrations. As a result, negative feedback regulation of glycolysis by [ATP] is established, allowing the rate of ATP production to adjust to the energy demands of the cell and thereby stabilizing [ATP] under varying rates of ATP consumption. Importantly, allosteric inhibition of PFK by ATP alone was insufficient to provide negative feedback regulation of glycolysis via [ATP]. The contribution of the adenylate kinase reaction to ATP production appears to be negligible. Also, due to the presence of adenylate kinase in cells, energy metabolism is regulated not by the absolute concentration of ATP, but by the energy charge or the ratio of [ATP] to the sum of [ATP], [ADP], and [AMP].

## 1. Introduction

Adenylate kinase (AK) or ATP/AMP phosphotransferase (EC 2.7.3.4) is an enzyme that catalyzes the following reversible reaction:

AMP + ATP ↔ 2 ADP(1)

By converting AMP to ADP, AK provides a functional link between purine metabolism and energy metabolism. This enzyme is ubiquitous, being present in all living organisms and all cell types and tissues. In eukaryotes, nine AK isoenzymes have been identified, differing in tissue distribution, intracellular localization, kinetic properties, and substrate specificity [1,2].

The activity of AK in cells is remarkably high compared with the rate of ATP turnover in energy metabolism. In human erythrocytes, AK activity reaches 1.5–5.5 mol·h^−1^·L^−1^ of cells [3,4], whereas the ATP turnover rate is only on the order of a few mmol·h^−1^·L^−1^ of cells [5]. In mammalian skeletal muscle, AK activity reaches 7–20 mmol·s^−1^·kg^−1^ of tissue [6,7,8,9], while maximal ATP turnover reaches only up to 3 mmol·s^−1^·kg^−1^ of tissue [10,11,12,13,14]. The equilibrium constant of the AK reaction is close to unity [15,16,17].

(2) $$K=\frac{\left[ATP\right][AMP]}{{\left[ADP\right]}^{2}}\approx 1$$

Owing to the high AK activity, the AK reaction is expected to be close to equilibrium in cells. Indeed, the ratios between the concentrations of ATP, ADP, and AMP are close to thermodynamic equilibrium in a wide variety of cells and tissues across different species [4,18,19,20]. Moreover, the data presented in [11,21,22] demonstrate that AK equilibrium is maintained even in actively contracting skeletal muscle.

Deficiency of AK activity can lead to serious pathologies and death of the organism [1,2,3,4].

Because the AK reaction converts two ADP molecules into ATP and AMP, it has been proposed that AK may serve as an additional source of ATP production in cells, particularly during transient or non-steady-state conditions [14,23,24]. However, to the best of our knowledge, the contribution of the AK reaction to ATP production in cells has not been quantitatively evaluated.

On the other hand, AK influences the concentration of AMP, which is a powerful allosteric regulator of a number of key metabolic enzymes, such as phosphofructokinase, glycogen phosphorylase, and AMP-activated protein kinase [23,25,26,27], and may play an important role in the regulation of energy metabolism and homeostasis in cells. At the same time, assessments of the role of the AK reaction in the regulation of cellular metabolism are rather descriptive in nature, and we are not aware of any studies that provide a quantitative assessment of this role.

The next section (“General Considerations”) provides a detailed discussion of the principles underlying the organization of cellular energy metabolism. It outlines the conditions under which the regulation of energy metabolism ensures ATP production in accordance with cellular demands, as well as stabilization of intracellular ATP concentration and energy charge. This regulation is possible only if the steady-state rate of ATP production decreases with increasing ATP concentration within the physiological [ATP] range.

In the Results section, we then use mathematical modeling to demonstrate that a highly active (near-equilibrium) AK reaction enables the regulation of glycolysis and thus ensures appropriate control of ATP production as described in the “General Considerations” section. We focus on cells in which glycolysis represents the primary source of ATP production, including erythrocytes and fast-twitch (anaerobic) skeletal muscle fibers of mammals [5,28,29,30,31]. We also quantitatively assessed the contribution of the AK reaction to ATP production. Our results demonstrate that AK plays a fundamental role in regulating the rate of ATP production via glycolysis. At the same time, the contribution of the AK reaction to overall ATP production is negligible. Thus, the AK reaction serves as a key regulatory component of cellular energy metabolism but cannot be regarded as a significant source of ATP production.

## 2. General Considerations

### 2.1. The Energy Metabolism of the Cell Operates Predominantly Under Steady-State Conditions

In most cells, the concentration of ATP is relatively low compared with the rate of its consumption. Indeed, in stimulated skeletal muscle, the rate of ATP consumption can reach up to 3 mmol·s^−1^·kg^−1^ of tissue [10,11,12,13,14]. Given that the ATP concentration in skeletal muscle is approximately 5 mmol·kg^−1^ of tissue [10,11,31,32,33,34,35,36,37], it would decline to zero within a few seconds unless the rate of ATP production closely matched the rate of ATP consumption. Similarly, experiments in various cell cultures have shown that when oxidative phosphorylation is inhibited, the rate of lactate formation via glycolysis increases to 200–400 mmol·h^−1^·kg^−1^ of cells [38]—close to the rate of ATP formation and consumption in this case. Considering that the ATP concentration in these experiments was 1.5–2.5 mmol·kg^−1^ of cells, it is straightforward to estimate that intracellular ATP would be depleted in less than one minute if ATP production were to cease. Together, these estimates demonstrate that cellular energy metabolism almost invariably functions in a stable steady state, in which consumed ATP is immediately replenished by ATP-producing pathways. This also indicates that ATP cannot serve as an energy storage molecule, but rather functions as an energy carrier linking metabolic processes that generate ATP with those that consume it.

### 2.2. Stable Operation of Cellular Energy Metabolism Requires That the Rate of ATP Production Increase in Response to a Decrease in ATP Concentration

**Figure 1:**
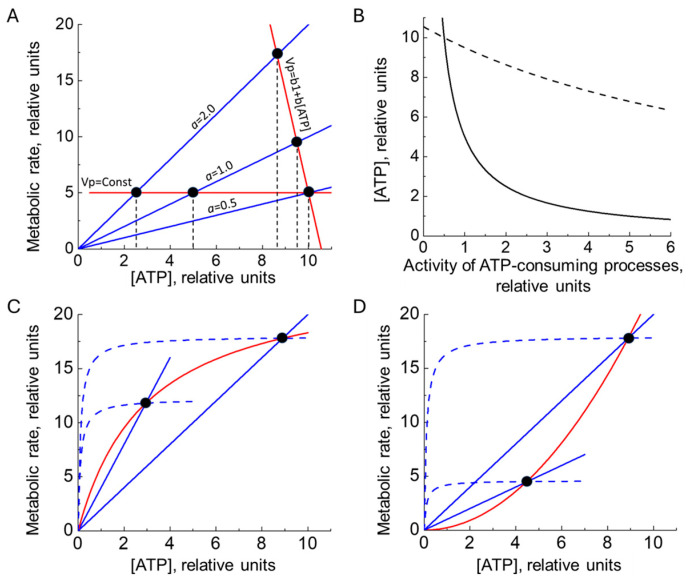
The influence of the activity of ATP-consuming processes on the steady-state concentration of ATP in energy metabolism, with different dependence of the rate of ATP production on [ATP]. (**A**) Linear dependence of the rate of ATP-consuming processes (V_c_) on [ATP] (V_c_ = *a*[ATP]) is shown by blue lines at the activity of ATP-consuming processes (*a*) equal to 0.5, 1.0, and 2.0 units. Red lines show ATP production at a constant rate (V_p_ = Const) and at a rate that decreases with increasing [ATP] (V_p_ = b1 + b[ATP]) at b1 = 95 and b = 9, and black dashed lines indicate steady-state ATP concentrations. (**B**) Dependence of the steady-state ATP concentration on the activity of ATP-consuming processes, calculated for two different dependences of the rate of ATP production on [ATP] shown in panel (**A**). The solid line is obtained for V_p_ = Const, and the dashed line is obtained for V_p_ = b1 + b[ATP]. (**C**,**D**) Red lines show two different versions of the dependence of ATP production rate on [ATP]. Blue lines show ATP consumption rate at linear (solid lines) and hyperbolic (dashed lines) dependence of ATP consumption rate on [ATP]. Black circles mark steady states of energy metabolism.

Consider a system in which the rate of ATP production is constant, whereas the rate of ATP consumption increases linearly with ATP concentration (Figure 1A). The intersection points of the ATP production and consumption graphs define the steady states of the system. Under these conditions, a stable steady state may, in principle, exist for any level of ATP-consuming activity. Stability is ensured by the fact that an increase in ATP concentration above its steady-state value causes the rate of ATP consumption to exceed the rate of production, thereby returning ATP concentration to the steady state. Conversely, a decrease in ATP concentration below the steady-state value results in ATP production exceeding consumption, again restoring the steady state. However, an increase in the activity of ATP-consuming processes in this scenario leads to a proportional decrease in the steady-state ATP concentration (Figure 1A,B). Such behavior can disrupt the independent operation of different ATP-consuming processes. Moreover, if the rate of ATP production remains constant, the steady-state rate of ATP consumption is also fixed and cannot adapt to changes in cellular energy demand (Figure 1A). This inability to match ATP production to consumption would inevitably impair normal cellular function.

The situation becomes even more problematic when the rate of ATP consumption depends only weakly on the ATP concentration. This corresponds to a hyperbolic dependence of the ATP consumption rate on [ATP], with a Michaelis constant for ATP that is much lower than the ATP concentration in the cell. In this case, a nonzero steady state can exist only if the maximal activity of ATP consumption is very close to the rate of ATP production. Any pronounced increase or decrease in the activity of ATP-consuming processes will lead to the loss of a nonzero steady state and to cell death.

The above analysis shows that an energy-producing system operating at a constant rate cannot support normal cellular function. Likewise, rational regulation of ATP concentration cannot be achieved if the rate of ATP production increases monotonically with ATP concentration. Two cases may be considered: one in which the slope of the ATP production rate decreases with increasing [ATP] (Figure 1C), and another in which it increases with increasing [ATP] (Figure 1D). In the first case, assuming a linear dependence of ATP consumption on ATP concentration, stable steady states may exist; however, the rate of ATP production decreases upon the activation of ATP-consuming processes (Figure 1C), which clearly contradicts the fundamental logic of metabolic regulation. When ATP consumption follows a hyperbolic dependence on [ATP], nonzero steady states become unstable. Specifically, a random increase in [ATP] above the steady-state level causes ATP production to exceed consumption (Figure 1C), leading to further ATP accumulation and loss of the steady state. Conversely, a random decrease in [ATP] results in ATP consumption exceeding production, ultimately driving ATP concentration—and consequently both production and consumption rates—toward zero. If the slope of the ATP production rate increases with increasing [ATP] (Figure 1D), all steady states are unstable.

The rate of ATP consumption should be dictated by the physiological tasks of the cell, rather than being constrained by ATP production. For ATP-consuming processes to operate independently, the intracellular ATP concentration must be effectively stabilized. Under such conditions, activation or inhibition of any individual ATP-consuming pathway does not alter ATP concentration and therefore does not interfere with the function of other ATP consumers. Experimental studies in various cells and tissues have indeed demonstrated stabilization of ATP levels despite changes in metabolic activity [11,12,13,38,39,40,41,42,43,44].

How, then, should the dependence of ATP production rate on [ATP] be structured to simultaneously ensure (i) adjustment of ATP production to changes in consumption and (ii) stabilization of ATP concentration? Based on the considerations above, only one solution remains: a negative slope in the dependence of ATP production rate on [ATP] within the physiological ATP range (Figure 1A). In this case, an increase in ATP consumption leads to a decrease in ATP concentration, which in turn stimulates ATP production, thereby restoring balance between production and consumption. The intersections of the ATP production and consumption graphs, where the rates of ATP production and consumption are equal to each other, define stable steady states of energy metabolism.

### 2.3. The ATP Concentration Stabilization Coefficient

To assess the quality of [ATP] stabilization under these conditions, consider the following mathematical formulation. Let the rates of ATP consumption (V_c_) and production (V_p_) be described by the linear functions of [ATP]

(3) $$V_{c}=a[ATP]$$

(4) $$V_{p}=b\left[ATP\right]+b_{1}$$

where parameters *a* and b_1_ are positive and parameter b is negative. The steady-state ATP concentration ([ATP]_ST_) is then given by

(5) $${\left[ATP\right]}_{ST}=\frac{b_{1}}{a-b}$$

To quantify the influence of ATP-consuming activity (*a*) on the steady-state ATP concentration, we employ the formalism of Metabolic Control Analysis [45,46,47] and define the corresponding control coefficient:

(6) $$C=\frac{d{\left[ATP\right]}_{ST}}{da}\cdot \frac{a}{{\left[ATP\right]}_{ST}}$$

Since

(7) $$\frac{d{\left[ATP\right]}_{ST}}{da}=\frac{d\left(\frac{b_{1}}{a-b}\right)}{da}=-\frac{b_{1}}{{\left(a-b\right)}^{2}}$$

it follows that

(8) $$C=-\frac{b_{1}}{{\left(a-b\right)}^{2}}\frac{a\left(a-b\right)}{b_{1}}=\frac{a}{b-a}$$

The smaller the control coefficient, the weaker the influence of ATP-consuming activity on the steady-state ATP concentration, and thus the more effective the stabilization of [ATP]. Notably, increasing the absolute value of the negative slope (b) reduces the magnitude of the control coefficient. We therefore introduce the ATP concentration stabilization coefficient, defined as the negative inverse of the control coefficient:

(9) $$Q=-\frac{1}{C}=-\frac{b-a}{a}=1-\frac{b}{a}$$

The negative sign ensures that the stabilization coefficient is positive when the slope of the ATP production graph is negative. As follows from this expression, the steeper the decrease in ATP production rate with increasing ATP concentration, the greater the stabilization of ATP concentration.

Figure 1B illustrates that when ATP production is constant (b = 0), changes in ATP-consuming activity lead to nearly proportional inverse changes in steady-state ATP concentration (Q = 1). In contrast, when the ATP production rate decreases with increasing [ATP] (b = −9), the ATP concentration varies much less in response to identical changes in ATP consumption (Q = 10 at *a* = 1; Figure 1B). Accordingly, the relative slopes of the curves ((*d*[ATP]/*da*)/[ATP]) in Figure 1B differ by an order of magnitude at *a* = 1.

In summary, these general considerations indicate that a universal principle of cellular energy regulation is the stabilization of ATP concentration through negative feedback control of ATP production. Specifically, the rate of ATP production must decrease steeply with increasing ATP concentration within the physiological range. Such regulation is required for all ATP-producing systems in the cell, including both glycolysis and oxidative phosphorylation. Thus, a graph of the steady-state dependence of the ATP production rate on [ATP] must contain a pronounced descending branch at physiological ATP levels, reflecting negative feedback regulation by ATP.

In the following sections, we use mathematical modeling to analyze the molecular mechanisms underlying this feedback in biological systems in which glycolysis is the sole or dominant source of ATP, namely human erythrocytes and fast-twitch (white) skeletal muscle fibers. We demonstrate that the adenylate kinase reaction plays a central role in establishing this feedback. At the same time, we show that the contribution of the AK reaction to ATP production is minor. Finally, we demonstrate that the AK reaction enables regulation of ATP production to be robust with respect to intercellular variability in adenine nucleotide concentrations.

## 3. Results

### 3.1. Parameters of the Normal Steady State in the Models

Our models provide a good description of the normal physiological steady state of energy metabolism in both human erythrocytes and resting mammalian skeletal muscle. In both models, the concentrations of ATP, ADP, AMP, and G6P lie within the range of available experimental data (Table 1). The steady-state value of the PFK reaction rate (V_PFK_) in the erythrocyte model, corresponding to the rate of glucose consumption in these cells, also falls within the experimentally observed range (Table 1). Similarly, the rate of ATP production in the resting mammalian skeletal muscle model lies within the range of experimental data (Table 1).

### 3.2. Metabolic Interactions Underlying Negative Feedback Regulation of Glycolysis by ATP

**Figure 2:**
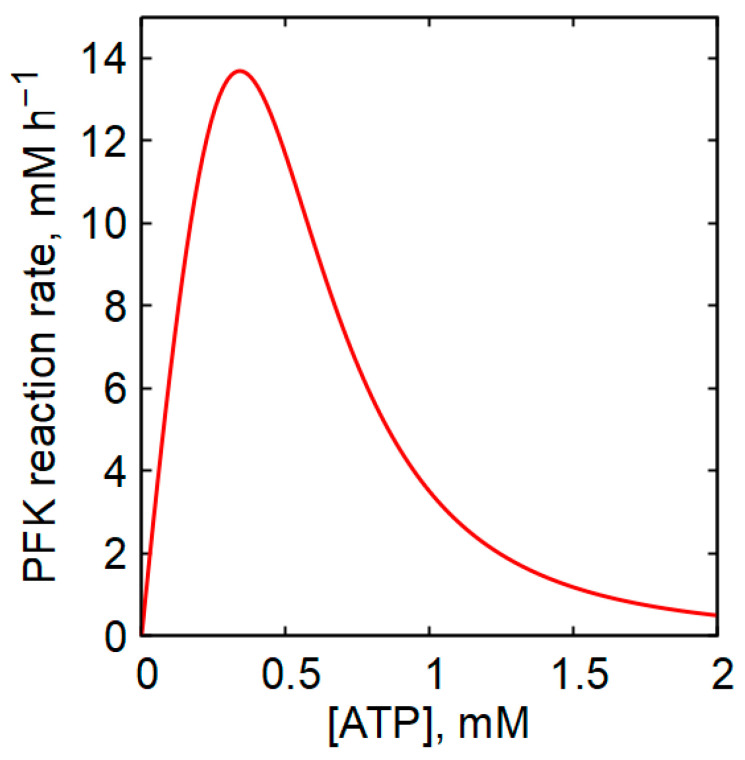
The dependence of the PFK reaction rate on [ATP], calculated using Equation (28) at A_PFK_ = 380 mM h^−1^ and concentrations of Pi and F6P equal to 1 mM and 25 µM, respectively. Such concentrations are typical for human erythrocytes [51].

**Figure 3:**
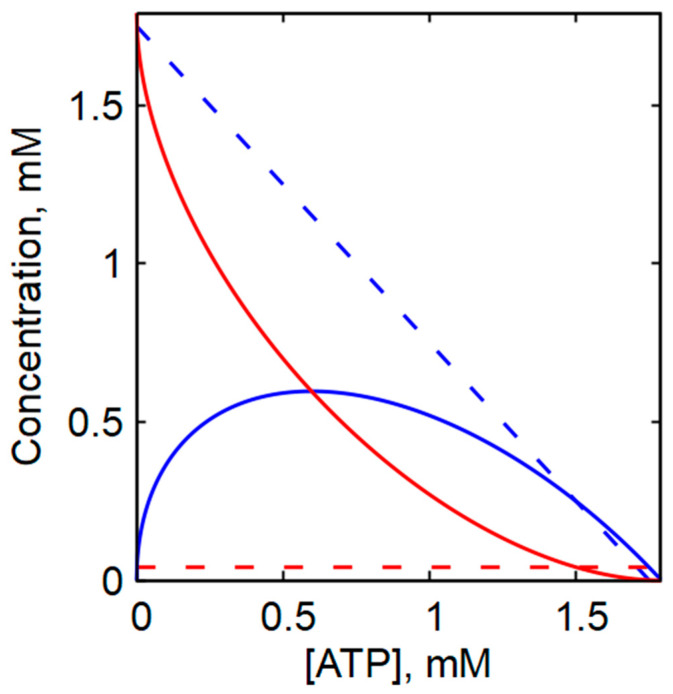
The influence of AK equilibrium on the ratio between the concentrations of ATP, ADP, and AMP in the cell. The dependence of steady-state concentrations of ADP (blue lines) and AMP (red lines) on the concentration of ATP in the presence of AK equilibrium (solid lines) and its absence (dashed lines), obtained with an adenine nucleotide pool value of 1.79 mM. In the absence of AK equilibrium, the AMP concentration was 40 μM.

It is generally accepted that the decrease in glycolytic rate with increasing intracellular ATP concentration is primarily due to the fact that ATP acts as a strong allosteric inhibitor of phosphofructokinase (PFK) [26,48,49,50]. The equation describing the PFK reaction rate used in our model predicts that at substrate concentrations typical of human erythrocytes, the reaction rate decreases sharply as [ATP] increases (Figure 2). However, in the presence of highly active adenylate kinase (AK), changes in ATP concentration are necessarily accompanied by substantial changes in ADP and, in particular, AMP concentrations, in accordance with Equation (2) (Figure 3). Figure 3 illustrates the dependence of the steady-state ADP and AMP concentrations on ATP concentration under conditions of a constant adenine nucleotide pool (A = [ATP] + [ADP] + [AMP]), in both the presence and absence of AK equilibrium. As shown in Figure 3, a decrease in ATP concentration within the physiological range leads to an increase in ADP concentration and, when AK equilibrium is present, to a pronounced increase in AMP concentration. Although the absolute concentration of AMP remains much lower than that of ADP, the relative increase in AMP concentration is markedly greater than the relative increase in ADP or the relative decrease in ATP. In the presence of AK equilibrium and a constant adenine nucleotide pool, the steady-state concentration of AMP becomes a quadratic function of the ATP concentration (Supplementary Materials, Equations (S1)–(S12)):

(10) $$[AMP]\approx A{\left(1-\frac{\left[ATP\right]}{A}\right)}^{2}$$

Thus, even small changes in ATP concentration result in large changes in AMP concentration. The strong dependence of [AMP] on [ATP] makes AMP an effective intracellular indicator of ATP fluctuations. Because AMP is a strong activator of PFK [26,48,49,50], these changes can profoundly influence the dependence of the PFK reaction rate—and consequently the glycolytic rate—on the ATP concentration.

In contrast, in the absence of AK, decreases in ATP concentration are accompanied by proportional accumulation of ADP, whereas the AMP concentration remains constant (Figure 3).

**Figure 4:**
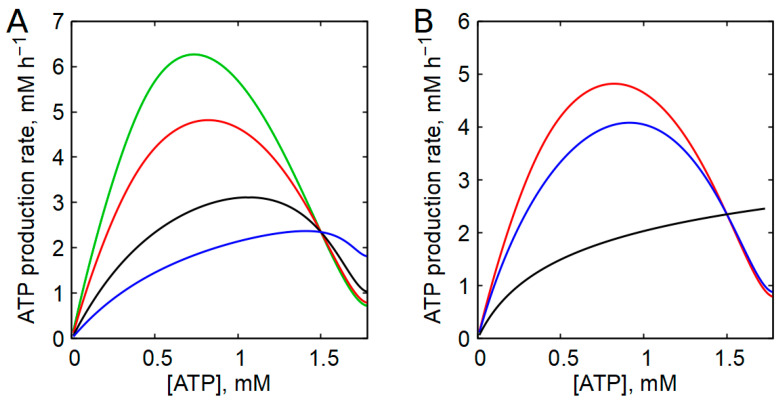
The effect of the inhibition constant of HK by G6P (**A**) and the allosteric regulation of PFK by ATP and AMP (**B**) on the dependence of the steady-state rate of glycolysis on [ATP] in the model with erythrocyte parameters. (**A**) Curves were obtained with the following pairs of HK inhibition constant for G6P and HK activity (A_HK_): green—0.55 µM and 102 mM h^−1^; red—5.5 µM and 12 mM h^−1^ (these values were considered normal); black—27.5 µM and 3.4 mM h^−1^; blue—220 µM and 2.2 mM h^−1^. For each value of the inhibition constant, the value of the HK activity was adjusted so that the curve passed through the point with physiologically normal values of the glycolysis rate and [ATP]. (**B**) Red, blue, and black curves were obtained with normal values of the parameters in the model, with the exclusion of the inhibition of PFK by ATP, and with the exclusion of the AK equilibrium from the model, respectively.

It should also be noted that effective regulation of glycolytic flux cannot be achieved solely through modulation of PFK activity, because the first reaction of the glycolytic pathway, catalyzed by hexokinase (HK), is irreversible. Therefore, coordination between HK and PFK reaction rates is required. This coordination is achieved through feedback inhibition of HK by its product, glucose-6-phosphate (G6P). When the PFK reaction rate decreases, G6P accumulates, inhibiting HK and thereby aligning the rates of the two reactions. As a result, the overall dependence of the glycolytic rate on ATP concentration is determined by the coupled regulation of HK and PFK, and the inhibition constant of HK for G6P strongly influences the shape of this dependence. Figure 4A shows the steady-state dependence of the ATP production rate on the ATP concentration in the erythrocyte model for different values of the HK inhibition constant for G6P. Similar results were obtained earlier with a different, more primitive model of human erythrocyte glycolysis [52]. Reported values of this constant span a wide range, from 2.5 to 70 μM [53,54,55,56,57]. The best agreement with experimental data obtained for human erythrocytes, in terms of both the steepness of the descending branch and the maximal glycolytic rate [42,58,59], was achieved with an inhibition constant of 5.5 μM and an HK activity of 12 mM h^−1^ (Supplementary Materials, Figure S1). These parameter values correspond to the red curve in Figure 4.

The effects of several additional model parameters on the steady-state dependence of glycolytic rate on [ATP] are shown in the Supplementary Materials, Figure S2. The maximal level of the curve is proportional to HK activity, which is expected because HK exhibits the lowest activity among glycolytic enzymes [51]. Variation in PFK activity has only a minor effect on the curve. In contrast, a significant increase in the parameter K_PFK3_ in Equation (28), corresponding to a decrease in PFK activation by AMP, leads to the disappearance of the descending branch of the glycolytic rate dependence on [ATP] (Supplementary Materials, Figure S2C).

### 3.3. Influence of Allosteric Regulation of PFK by AMP and ATP on the Shape of the Steady-State Dependence of the Glycolysis Rate on [ATP]

We next examined the relative contributions of ATP- and AMP-dependent allosteric regulation of PFK to the overall shape of the dependence of the glycolysis rate on the ATP concentration. Removal of AK equilibrium from the model, which effectively eliminates AMP-dependent regulation of PFK, qualitatively altered the dependence of the glycolysis rate on [ATP], converting it into a monotonically increasing function of ATP concentration (Figure 4B).

As shown above (Figure 1), such a dependence is incompatible with proper regulation of ATP production in accordance with cellular energy demands. By contrast, elimination of ATP-mediated inhibition of PFK from the equation for the PFK reaction rate (Equation (28)) resulted in only minor quantitative changes in the dependence of the glycolysis rate on [ATP] (Figure 4B). These results indicate that the shape of the glycolysis rate’s dependence on ATP concentration is determined primarily by AMP-dependent regulation of PFK, which in turn relies on the maintenance of AK equilibrium.

### 3.4. Influence of Adenylate Kinase Equilibrium on Steady-State Energy Metabolism

**Figure 5:**
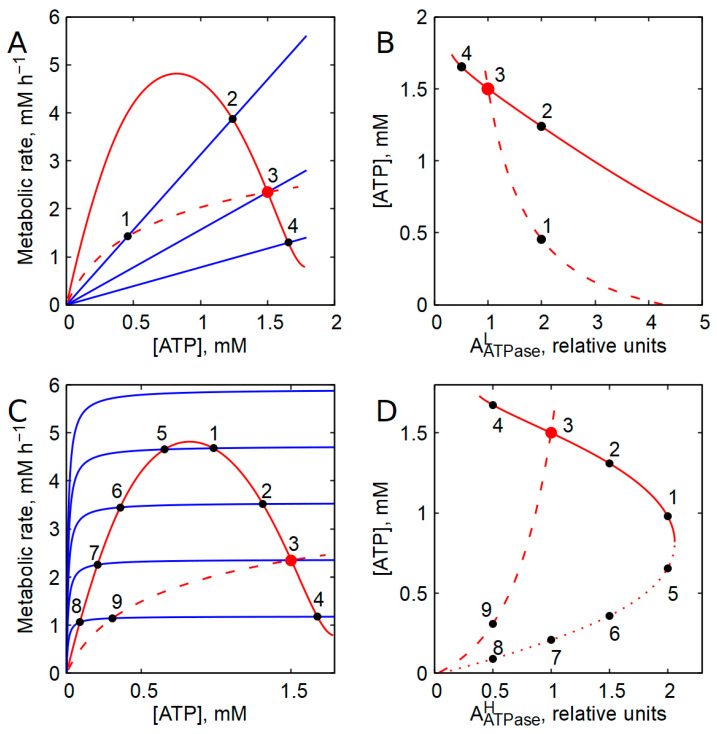
The influence of the AK equilibrium on the regulation of energy metabolism in human erythrocytes. (**A**) Red lines show the steady-state dependences of the rate of ATP production in glycolysis on [ATP] in the presence of AK equilibrium (solid line) and the absence of AK (dashed line). The blue lines show the dependence of the rate of ATP consumption on [ATP] at three different slope values equal to 0.5, 1, and 2 times the normal physiological slope value. (**B**) Stationary dependences of ATP concentration on the activity of ATP-consuming processes, obtained with a linear dependence of the rate of ATP consumption on [ATP] in the presence of AK equilibrium (solid line) and the absence of AK (dashed line). (**C**) Red lines correspond to the red lines in panel (**A**). The blue lines show the hyperbolic dependence of the rate of ATP consumption on [ATP] at a Michaelis constant of 10 μM and different values of the maximal rate of ATP consumption, which are equal to 0.5, 1, 1.5, 2, and 2.5 times the normal physiological rate of ATP consumption. (**D**) Stationary dependences of the ATP concentration on the activity of ATP-consuming processes, obtained with a hyperbolic dependence of the rate of ATP consumption on [ATP] with a Michaelis constant value equal to 10 μM in the presence of AK equilibrium (solid and dotted lines) and the absence of AK (dashed line). In panel (**D**), the dotted and dashed lines correspond to unstable steady states. Black and red circles mark individual stationary states. The same numbers next to the steady states in the left and right panels indicate the same steady states. Red circles indicate physiologically normal steady states. The results were obtained at an adenine nucleotide pool value of 1.79 mM.

In the presence of AK equilibrium, the steady-state dependence of the glycolytic rate on ATP concentration in the erythrocyte model exhibits a bell-shaped profile, with a steeply descending branch in the region of physiological ATP concentrations (Figure 5A). Similar curves were obtained with other models of glycolysis and energy metabolism in human erythrocytes [42,52,60,61]. This dependence is in good agreement with experimental data on the dependence of the glycolysis rate on the concentration of ATP obtained in intact human erythrocytes [42,58,59] (Supplementary Materials, Figure S1), as well as in erythrocytes from sheep, mice, dogs, and cows [62,63]. We refer to this dependence (curve) as the “glycolysis characteristic” [42,52], as it defines key functional properties of glycolysis, including the maximal rate of ATP production and the capacity to stabilize ATP concentration. The height of the bell-shaped curve reflects the maximal glycolytic ATP production rate, whereas the steepness of the descending branch determines the efficiency of ATP stabilization in response to changes in ATP consumption. The steeper this descending branch, the stronger the stabilization of ATP concentration. The characteristic of glycolysis provides a clear idea of how the glycolysis rate and the concentration of ATP in the cell change with changes in parameters of energy metabolism, such as the activity of ATP-consuming processes.

Figure 5A illustrates the interaction between glycolysis and ATP-consuming processes in erythrocytes, assuming a linear dependence of the ATP consumption rate on ATP concentration. The intersections of ATP production and consumption graphs define the steady states of cellular energy metabolism. In the presence of AK equilibrium, a twofold change in ATP-consuming activity results in only an approximately 10% change in ATP concentration (Figure 5A,B). Thus, AK equilibrium ensures both the existence of stable steady states and robust ATP stabilization over a broad range of ATP consumption rates. In the absence of AK, the dependence of the glycolysis rate on the ATP concentration becomes a monotonically increasing function of [ATP] (Figure 5A). Under these conditions, stable steady states may still exist for linear ATP consumption kinetics, but ATP concentration is not stabilized (Figure 5B). Moreover, steady-state rates of ATP production and consumption cannot be substantially increased beyond physiological values, a situation incompatible with sustained cellular viability.

When ATP consumption follows a hyperbolic dependence on ATP concentration, two steady states may coexist for a given level of ATP-consuming activity (Figure 5C,D). Steady states located on the descending branch of the glycolysis characteristic curve (points 1–4 in Figure 5C) are stable, whereas those on the ascending branch (points 5–8) are unstable. In these states, perturbations in ATP concentration drive the system either toward a higher-ATP stable state or toward the collapse of ATP concentration to zero. When ATP-consuming activity exceeds the maximum supported by the glycolysis characteristic, nonzero steady states disappear entirely. In this case, the stationary dependence of the ATP concentration on the activity of ATP-consuming processes contains a branch with the stable steady states demonstrating ATP stabilization, a branch corresponding to unstable steady states, and a bifurcation point at the maximal acceptable activity of ATP-consuming processes (Figure 5D).

In models lacking AK equilibrium, stable, nonzero steady states are generally absent under hyperbolic ATP consumption kinetics (Figure 5C,D).

Similar results were obtained using parameters representative of mammalian skeletal muscle (Supplementary Materials, Figure S3).

### 3.5. Contribution of the Adenylate Kinase Reaction to ATP Production

The adenylate kinase reaction redistributes adenine nucleotides within the cellular pool, producing one ATP and one AMP molecule from two ADP molecules. Because no net free energy is generated, this reaction cannot serve as a sustained source of ATP. In steady state, ATP formation via AK is exactly balanced by the ATP consumption required to reconvert AMP to ADP, resulting in a net zero energy balance.

**Figure 6:**
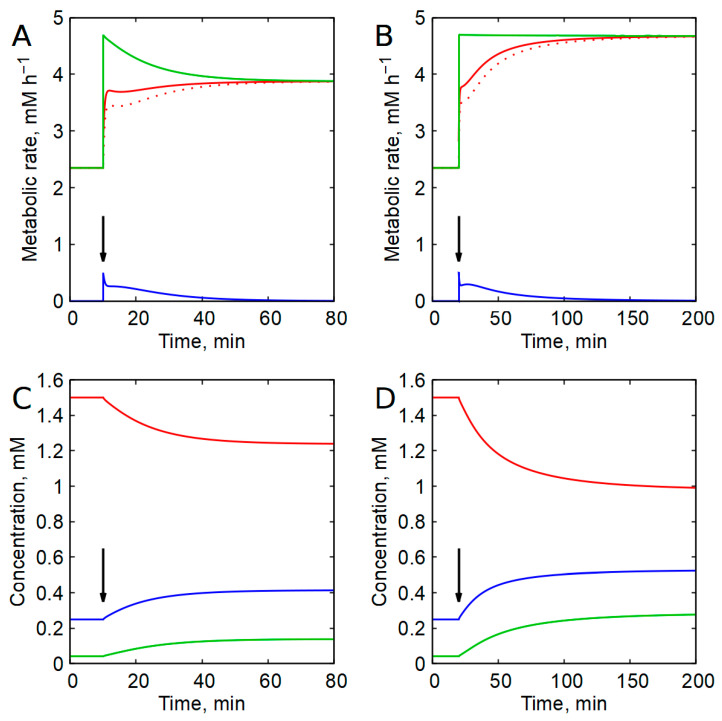
Kinetics of adenine nucleotide levels and the contribution of the AK reaction to the rate of ATP production in the model of human erythrocytes after an instantaneous twofold increase in the activity of ATP-consuming processes in the presence of AK equilibrium. (**A**) Kinetics of the rate of ATP consumption (green line), the total rate of ATP production (red line), the total rate of ATP production minus the AK reaction rate (red dotted line), and the rate of ATP production in the AK reaction (blue line) in erythrocytes obtained with a linear dependence of the rate of ATP consumption on [ATP]. (**B**) Kinetics of the rate of ATP consumption (green line), the total rate of ATP production (red line), the total rate of ATP production minus the AK reaction rate (red dotted line), and the rate of ATP production in the AK reaction (blue line) in erythrocytes with a hyperbolic dependence of the rate of ATP consumption on [ATP] with a Michaelis constant equal to 10 μM. (**C**) Kinetics of [ATP] (red line), [ADP] (blue line), and [AMP] (green line) obtained with a linear dependence of the rate of ATP consumption on [ATP]. (**D**) Kinetics of [ATP] (red line), [ADP] (blue line), and [AMP] (green line) obtained with a hyperbolic dependence of the rate of ATP consumption on [ATP] with a Michaelis constant equal to 10 μM. The activity of ATP-consuming processes changed at the time indicated by the arrow from 1.57 mM h^−1^ to 3.24 mM h^−1^ in the case of a linear dependence and from 2.36 mM h^−1^ to 4.72 mM h^−1^ in the case of a hyperbolic dependence of the rate of ATP consumption on [ATP]. The results were obtained at an adenine nucleotide pool value of 1.79 mM.

Model analysis confirms this conclusion. AK can make only a limited and transient contribution to ATP production following abrupt increases in ATP consumption (Figure 6A,B). Such transients are associated with the temporary accumulation of AMP (Figure 6C,D), which reflects ATP formation in the AK reaction. For a twofold instantaneous increase in ATP-consuming activity, the AK contribution does not exceed approximately 18% of total ATP production, regardless of whether ATP consumption depends linearly or hyperbolically on ATP concentration (Figure 6A,B).

Comparable results were obtained in skeletal muscle models (Supplementary Materials, Figure S4). The data presented in Figure S4 were obtained without explicitly accounting for ATP production in the creatine kinase (CK) reaction. However, in skeletal muscles, this reaction significantly contributes to ATP production during transient processes [24]. When the CK reaction is included in the model, the relative contribution of the AK reaction to ATP production becomes even smaller and does not exceed 1% of the total ATP production rate (Supplementary Materials, Equations (S13)–(S41), Figure S5).

Experimental data from contracting skeletal muscle, where ATP consumption increases by an order of magnitude or more, indicate that ATP concentration remains well stabilized and AMP accumulation remains modest [11,13,43,44]. This further supports the conclusion that AK does not make a significant contribution to ATP production, even under high energetic demand.

### 3.6. Adenylate Kinase Equilibrium Ensures Regulation of Glycolysis by the Relative Concentrations of ATP and AMP

Cellular energy metabolism is not strictly tied to any specific ATP concentration. Different cell types and tissues maintain different levels of ATP, specific to the particular cells or tissues [64]. Moreover, these cell- or tissue-specific ATP levels have different values in cells and tissues of different individuals of the same species. For example, the concentration of ATP in human erythrocytes varies more than twofold between different individuals [65,66]. Nevertheless, under physiological conditions, most cells maintain a ratio of ATP, ADP, and AMP concentrations close to the following [4,18,19,20]:

[ATP] : [ADP] : [AMP] = 100 : 10 : 1(11)

About 50 years ago, D.E. Atkinson suggested that cells do not maintain an absolute concentration of ATP, but rather an energy charge, which is determined by the following expression [67]:

(12) $$\varphi =\frac{\left[ATP\right]+0.5[ADP]}{\left[ATP\right]+\left[ADP\right]+[AMP]}$$

**Figure 7:**
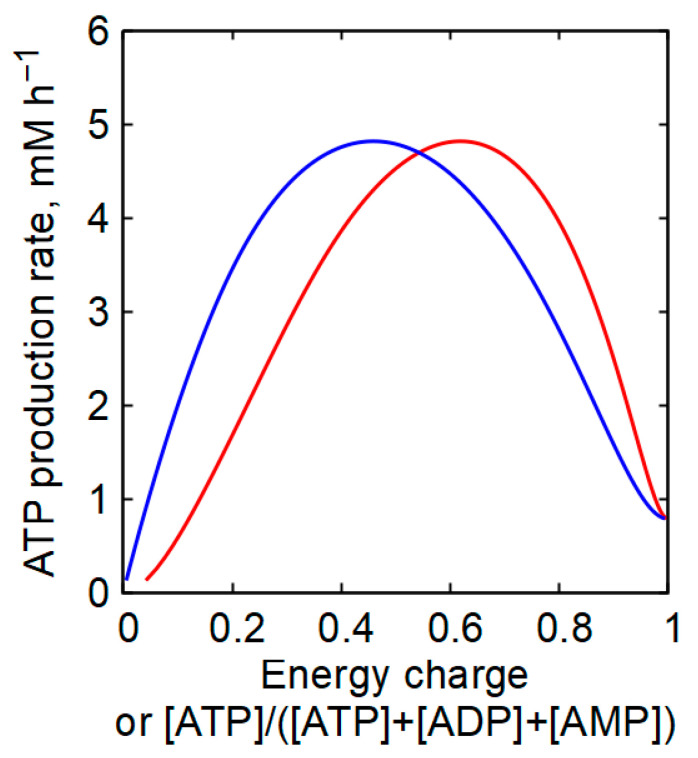
Dependence of the rate of ATP production in glycolysis on the energy charge (red line) and on the relative concentration of ATP ([ATP]/([ATP] + [ADP] + [AMP]) (blue line) in human erythrocytes in the presence of AK equilibrium. The results were obtained with an adenine nucleotide pool value equal to 1.79 mM.

For the ratio in Equation (11), the energy charge equals 0.95. Under physiological conditions, the energy charge closely approximates the relative ATP concentration (the ratio of [ATP] to the pool of adenine nucleotides), and the dependence of the glycolytic rate on the energy charge closely mirrors its dependence on the relative ATP concentration (Figure 7). A similar result was observed in skeletal muscle models (Supplementary Materials, Figure S6).

**Figure 8:**
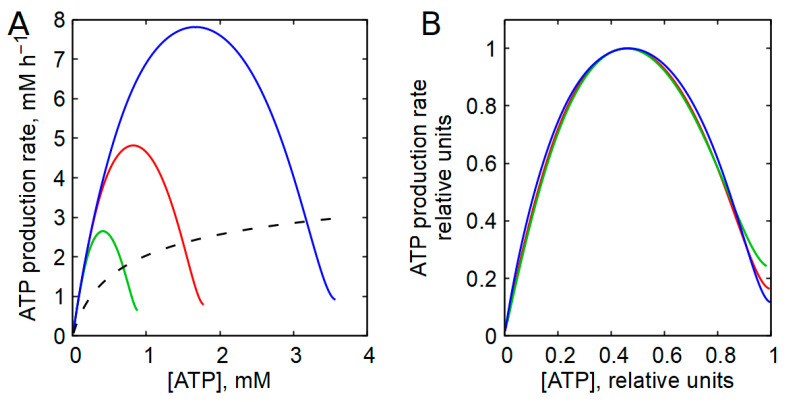
The influence of the AK equilibrium on the steady-state dependence of the glycolysis rate on the ATP concentration in erythrocytes at different values of the adenine nucleotide pool. (**A**) Solid lines show the steady-state dependences of the rate of ATP production in glycolysis on the absolute concentration of ATP, obtained for adenine nucleotide pool values equal to 3.58 mM (blue line), 1.79 mM (red line), and 0.9 mM (green line) in the presence of AK equilibrium. Dashed line shows the steady-state dependence of the rate of ATP production in glycolysis on the absolute concentration of ATP, obtained in the absence of AK equilibrium at constant AMP concentration of 40 μM. (**B**) Graphs of the bell-shaped curves shown in panel (**A**), obtained in the presence of AK equilibrium, after normalization to the maximum point.

While absolute ATP concentrations in the same cell type may vary more than twofold among individuals, energy charge values typically vary by less than 25% [68]. This indicates that the adenine nucleotide pool size determines the absolute ATP concentration, whereas metabolic regulation determines the energy charge. Consequently, energy metabolism is regulated primarily by relative, rather than absolute, nucleotide concentrations. This regulation is ensured by AK. In the presence of AK equilibrium, changes in the adenine nucleotide pool lead to proportional changes in ATP and AMP concentrations. Since ATP is an inhibitor of the PFK reaction, and AMP is an activator, the rate of the PFK reaction is largely determined by the ratio of the concentrations of AMP and ATP (Equation (28)). And the ratio of [AMP] to [ATP] does not depend on the pool of adenine nucleotides, but on the relative concentration of ATP (Supplementary Materials, Equation (S12)), which is normally maintained in cells at the level of 90% of the pool of adenine nucleotides (Equation (11)). As a result, the ratio of [AMP] to [ATP] practically does not change when the total pool of adenine nucleotides changes, and the glycolysis characteristic retains its shape across different pool sizes (Figure 8A). After normalization to their maxima, glycolysis characteristics obtained at different pool sizes nearly coincide (Figure 8B), in agreement with experimental observations in erythrocytes from different individuals and species [42,59,62,63].

In contrast, in the absence of AK, the glycolytic rate depends strongly on the absolute ATP concentration (Figure 8A).

Similar results were obtained in skeletal muscle models (Supplementary Materials, Figure S7).

## 4. Discussion

The discussion presented in the General Considerations section demonstrated that regulation of ATP production in accordance with cellular energy demand can be achieved only if the steady-state rate of ATP production decreases as the ATP concentration increases. In cells where glycolysis is the primary source of ATP, this requirement implies that the dependence of the glycolytic rate on [ATP] must contain a descending branch within the physiological range of ATP concentrations.

Using relatively simple mathematical models, we examined the extent to which AK contributes to the formation of this descending branch in human erythrocytes and mammalian white skeletal muscle. Key assumptions of these models include conservation of the adenine nucleotide pool and maintenance of equilibrium ratios among adenine nucleotides (ATP, ADP, and AMP). These assumptions appear physiologically reasonable. The characteristic time scale for changes in the adenine nucleotide pool in erythrocytes is on the order of tens of hours [69], which is much longer than the characteristic time scales of energy metabolism. In skeletal muscle, the adenine nucleotide pool changes only minimally, even under high ATPase loads [11,21,22]. With regard to AK equilibrium, the high activity of AK relative to metabolic fluxes enables maintenance of near-equilibrium conditions in diverse cell types and tissues [3,4,6,7,8,9,18,19,20], including contracting skeletal muscle [11,21,22].

Model analysis revealed that the descending branch of the steady-state dependence of the glycolytic rate on ATP concentration is formed primarily as a consequence of the function of highly active AK. Thus, AK plays a central role in regulating glycolytic flux by controlling intracellular AMP levels. The descending branch of the steady-state dependence of glycolytic rate on ATP concentration (hereafter referred to as the glycolysis characteristic) represents negative feedback regulation of ATP production by ATP itself. This descending branch is essential for proper control of the ATP production rate and for stabilization of the intracellular ATP concentration. Our results demonstrate that, in the absence of AK, allosteric inhibition of PFK by ATP alone is insufficient to generate a descending branch of the glycolysis characteristic curve (Figure 4B, Figure 5 and Figure S3). These findings are far from intuitive and substantially expand current understanding of glycolytic regulation.

The steep decline in glycolytic flux with increasing ATP concentration arises primarily because AK amplifies small increases in [ATP] within the physiological range into large decreases in AMP concentration, a potent activator of PFK (Figure 3). In addition, the formation of this steep descending branch requires coordinated interaction between PFK and HK, mediated by G6P (Figure 4A). Taken together, these results indicate that negative feedback regulation of glycolysis by ATP emerges from the combined action of AK, PFK, and HK. This mechanism operates in both erythrocytes and mammalian white (anaerobic) skeletal muscle (Figure 5 and Figure S3).

In the presence of AK, the glycolysis characteristic curve exhibits a bell-shaped profile, with a pronounced descending branch within the physiological ATP range (Figure 5 and Figure S3). The existence of this descending branch ensures acceleration of ATP production in response to increased ATP consumption. The steeper the descending branch, the more effectively ATP concentration is stabilized against fluctuations in ATP-consuming activity. The characteristic of the ascending branch reflects the intrinsic biochemical structure of glycolysis. Because glycolysis begins with ATP-consuming reactions catalyzed by HK and PFK, the glycolytic flux must be zero when the ATP concentration or energy charge approaches zero. This constraint necessarily produces a curve with a maximum, of which only the descending branch is physiologically functional.

When ATP consumption displays a hyperbolic dependence on ATP concentration with a low Michaelis constant for ATP, abrupt loss of a nonzero steady state may occur when ATP consumption exceeds the maximal achievable rate of ATP production by glycolysis (Figure 5C,D and Figure S3C,D). Under these conditions, the only remaining steady state corresponds to complete ATP depletion.

Efficient stabilization of ATP concentration or energy charge—achieved through AK-dependent regulation of glycolytic flux—ensures functional independence of individual ATP-consuming processes within the cell. Activation or inhibition of a single ATP consumer then induces only minor changes in ATP concentration and therefore exerts minimal influence on other ATP-dependent processes.

In contrast, in the absence of AK, proper regulation of glycolytic flux and stabilization of ATP concentration cannot be achieved because the glycolysis characteristic curve lacks a descending branch (Figure 5 and Figure S3). Moreover, under these conditions, glycolytic flux cannot be substantially increased above its basal physiological level, implying that glycolysis cannot meet elevated energy demands. Thus, AK is essential for normal glycolytic function in cells and, actually, for cell viability.

Using the models, we also quantitatively evaluated the contribution of the AK reaction to ATP production in erythrocytes and mammalian white skeletal muscle cells. The AK reaction makes no net contribution to ATP production at steady state. The ATP produced by AK is exactly balanced by the ATP consumption required for AMP rephosphorylation, resulting in zero net ATP gain. AK may provide a small and transient contribution following abrupt changes in ATP consumption (Figure 6, Figures S4 and S5). However, contrary to earlier suggestions [14,23,24], this contribution does not significantly affect overall ATP production.

An additional important consequence of AK activity revealed by the model analysis is that cellular energy metabolism is governed primarily by relative rather than absolute adenine nucleotide concentrations, largely independently of total adenine nucleotide pool size (Figure 8 and Figure S7). While the pool size and absolute concentrations of adenine nucleotides may act as additional regulatory parameters, AK ensures regulation primarily through ratios such as [ATP]/([ATP] + [ADP] + [AMP]). Modulation of the adenine nucleotide pool size may therefore provide additional degrees of metabolic control, for example, improving stabilization of cell volume in human erythrocytes [61,68,70,71].

Based on these findings, the following conclusions can be drawn:

- The adenylate kinase reaction plays a fundamental role in the regulation of cellular energy metabolism.
- The contribution of adenylate kinase to ATP production is zero at steady state and negligible under physiologically significant transient conditions.
- In the presence of adenylate kinase, energy metabolism is regulated primarily by relative ATP levels or energy charge, rather than by absolute ATP concentration.

We believe that these conclusions apply broadly to eukaryotic cells. As mentioned above, highly active AK is present in all cells and tissues. The slow rate of change in the adenine nucleotide pool relative to metabolic fluxes follows from the basic requirements of metabolic stability [70]. Thus, the conditions under which AK plays a key role in regulating glycolytic flux are likely to be satisfied in all cells. However, in cells where glycolysis contributes little to ATP production, this regulatory mechanism may not play a major role in overall energy metabolism.

Finally, we predict that experimental validation of the main finding of this study—the central role of AK in regulating glycolytic flux—can be achieved using strong suppression of AK activity in human erythrocytes. Such suppression should disrupt glycolytic regulation and ATP stabilization. Under these conditions, an increase in ATPase activity (for example, by activating Na^+^/K^+^-ATPase through increased cell membrane permeability with amphotericin B [40,41]) should not cause glycolytic activation. As a result, ATP concentration should decrease significantly compared to control cells.

## 5. Methods (Description of Mathematical Models)

### 5.1. General Structure of the Models

In this study, we analyzed mathematical models of cellular energy metabolism in human erythrocytes and mammalian white (anaerobic) skeletal muscle. The models were developed based on our previous models of glycolysis and energy metabolism in human erythrocytes, published elsewhere [42,51,52,60,61,72]. The cited models describe the experimental data on the regulation of glycolysis and energy metabolism in human erythrocytes well. The models used in this study include glycolysis and ATP-consuming processes (ATPase activity). Each model was examined in two versions: with the adenylate kinase (AK) reaction present in equilibrium and without AK. In all cases, the model includes only the upper part of glycolysis, consisting of the first three reactions catalyzed by hexokinase (HK), glucose phosphate isomerase (GPI), and phosphofructokinase (PFK). These reactions fully determine the steady-state glycolytic flux. The influence of the pentose phosphate pathway and the 2,3-diphosphoglycerate shunt on ATP production was neglected. The concentrations of glucose, inorganic phosphate (Pi), and the total adenine nucleotide pool (A = [ATP] + [ADP] + [AMP]) were assumed to be constant (Table 1). This reduced model accurately reproduces the experimentally observed dependence of the steady-state glycolytic rate on ATP concentration in human erythrocytes [42]. This dependence represents a key characteristic of glycolysis, as it determines both the degree of ATP stabilization in response to changes in ATP consumption and the maximal attainable rate of ATP production.

### 5.2. Models Without Adenylate Kinase

The model without AK consists of two differential equations describing metabolite dynamics in the upper part of glycolysis and one differential equation for ATP concentration, under the assumptions of a constant adenine nucleotide pool and constant AMP concentration:

(13) $$\frac{d\left[G6P\right]}{dt}=V_{HK}-V_{PGI}$$

(14) $$\frac{d\left[F6P\right]}{dt}=V_{GPI}-V_{PFK}$$

(15) $$\frac{d\left[ATP\right]}{dt}=-V_{HK}+3V_{PFK}-V_{ATPase}$$

(16) $$A=\left[ATP\right]+\left[ADP\right]+\left[AMP\right]=const$$

(17) [AMP]=const

Here, G6P and F6P denote glucose-6-phosphate and fructose-6-phosphate, respectively. V_HK_, V_PGI_, V_PFK_, and V_ATPase_ represent the rates of the hexokinase, glucose phosphate isomerase, and phosphofructokinase reactions and the total rate of ATP consumption (rate of ATP-consuming processes), respectively. We take into account that ATP is consumed in glycolysis at a rate of V_HK_ + V_PFK_. We assume that the rates of ATP production in phosphoglycerate kinase (PGK) and pyruvate kinase (PK) reactions are equal to twice the rate of the PFK reaction, that is, V_PGK_ = V_PK_ = 2V_PFK_. Consequently, the total ATP production rate in the lower part of glycolysis is V_PGK_ + V_PK_ = 4V_PFK_. AMP concentration was fixed at its physiologically normal value for the corresponding cell type (Table 1).

### 5.3. Models with Adenylate Kinase in Equilibrium

The model incorporating AK reaction in equilibrium (Equation (2)) includes Equations (13) and (14), together with explicit equations for adenine nucleotide dynamics:

(18) $$\frac{d\left[ATP\right]}{dt}=-V_{HK}+3V_{PFK}-V_{ATPase}+V_{AK}$$

(19) $$\frac{d\left[ADP\right]}{dt}=V_{HK}-3V_{PFK}+V_{ATPase}-2V_{AK}$$

(20) $$\frac{d\left[AMP\right]}{dt}=V_{AK}$$

Here, V_AK_ denotes the rate of the adenylate kinase reaction. For convenience, we introduce a composite variable E = 2[ATP] + [ADP] for which the differential equation does not explicitly contain the AK reaction rate:

(21) $$\frac{dE}{dt}=-V_{HK}+3V_{PFK}-V_{ATPase}$$

Using the AK equilibrium condition (Equation (2)) and the constant adenine nucleotide pool constraint (Equation (16)), the concentrations of ATP, ADP, and AMP can be expressed as algebraic functions of (E):

(22) $$\left[ATP\right]=\frac{1}{2}\left(E+\frac{A-Q}{4K-1}\right)$$

(23) $$\left[ADP\right]=\frac{Q-A}{4K-1}$$

(24) $$\left[AMP\right]=\frac{1}{2}\left(2A-E+\frac{A-Q}{4K-1}\right)$$

Here,

(25) $$Q=\sqrt{A^{2}-2AE+E^{2}+8AEK-4E^{2}K}$$

Thus, the final model with AK consists of three differential Equations (13), (14) and (21) and algebraic expressions (22)–(25) for adenine nucleotide concentrations.

### 5.4. Equations for the Rates of Enzymatic Reactions of Glycolysis in Human Erythrocytes

Rate equations and parameter values for glycolytic enzymes in human erythrocytes, based on experimental data presented in the literature, were taken from [51,72].

The hexokinase reaction was modeled as irreversible and dependent on ATP and G6P concentrations, while glucose concentration was assumed constant:

(26) $$V_{HK}=A_{HK}\frac{\left[ATP\right]/K_{HK1}}{1+[ATP]/K_{HK1}+[G6P]/K_{HK2}}$$

Here, A_HK_ = 12 mM h^−1^, K_HK1_ = 1 mM, and K_HK2_ = 5.5 µM.

The GPI reaction was described as a reversible isomerization between G6P and F6P:

(27) $$V_{GPI}=A_{GPI}\frac{([G6P]-\left[F6P\right]K_{GPI1})/K_{GPI2}}{1+\frac{\left[G6P\right]}{K_{GPI2}}+\frac{\left[F6P\right]}{K_{GPI3}}}$$

Here, A_GPI_ = 360 mM h^−1^, K_GPI1_ = 3 mM, K_GPI2_ = 0.3 mM, and K_GPI3_ = 0.2 mM.

The PFK reaction was modeled as an irreversible reaction depending on ATP and F6P concentrations and incorporating allosteric activation by AMP and inhibition by ATP, as well as explicit dependence on inorganic phosphate (Pi) [73]:

(28) $$\begin{array}{c} V_{PFK}=A_{PFK}\left(1+\frac{\left[Pi\right]}{K_{PFK6}}\right)\cdot \frac{\left[F6P\right]}{\left[F6P\right]+K_{PFK1}}\cdot \frac{\left[ATP\right]}{\left[ATP\right]+K_{PFK2}}\\ \;\cdot \frac{\frac{1}{1+\left[AMP\right]/K_{PFK3}}+2\frac{\left[AMP\right]}{\left[AMP\right]+K_{PFK3}}}{1+10^{8}{\left(\frac{\left(1+\left[ATP\right]/K_{PFK4}\right)}{\left(1+\left[AMP\right]/K_{PFK3}\right)\left(1+\left[F6P\right]/K_{PFK5}\right)}\right)}^{4}} \end{array}$$

Here, A_PFK_ = 380 mM h^−1^, K_PFK1_ = 0.1 mM, K_PFK2_ = 2 mM, K_PFK3_ = 0.01 mM, K_PFK4_ = 0.195 mM, K_PFK5_ = 0.37 µM, and K_PFK6_ = 10 mM.

To eliminate ATP-mediated allosteric inhibition of PFK, ATP concentration in the inhibitory term (1 + [ATP]/K_PFK4_) was fixed at 1.5 mM, corresponding to its physiological value. In this case, the glycolysis rate at a physiologically normal concentration of ATP does not change, and there is no need to vary the model parameters.

Physiologically normal values of the metabolite concentrations, adenine nucleotide pool, and metabolic rates in the human erythrocyte model are given in Table 1.

**Table 1:** Normal physiological values of metabolite concentrations, adenine nucleotide pool, and metabolic rates in human erythrocytes and white skeletal muscles of mammals. The table presents the values of the parameters and variables in the model and the range of their experimental values (in brackets), taken from [51] for human erythrocytes and [74] for white skeletal muscles of mammals. The cited works contain a large pool of experimental data from the literature regarding the parameters of energy metabolism in human erythrocytes and mammalian skeletal muscles, respectively.

Parameter or Variable	Human Erythrocytes	Skeletal Muscles	Units
[ATP]	1500 (1070–1830)	4980 (3980–5740)	µM
[ADP]	250 (85–300)	390 (135–640)	µM
[AMP]	40 (10–50)	30 (14–98)	µM
A—Adenine nucleotide pool ([ATP] + [ADP] + [AMP])	1790	5400	µM
[G6P]	70.6 (20–110)	181 (59–516)	µM
[F6P]	23.1 (6–16)	60 (34–134)	µM
[Pi]—orthophosphate concentration	1000	2000 (580–11,500)	µM
V_PFK_—PFK reaction rate (metabolic flux in the upper part of glycolysis)	1.17 (0.6–1.46)	12.8	mM h^−1^
ATP production rate in glycolysis	2.34	25.6 (8–58)	mM h^−1^

### 5.5. Model Parameters for Skeletal Muscle

Glycolysis in resting skeletal muscle was modeled assuming glucose as the sole substrate, neglecting glycogen-derived fluxes. We also neglect the possible production of ATP in oxidative phosphorylation. The same kinetic equations for the enzymatic reaction rates as for erythrocytes were used, with different parameter values for enzyme activities, Pi concentration, and adenine nucleotide pool size (Table 1 and Table 2). Under physiological conditions, the model predicts a PFK flux of 12.8 mM h^−1^, corresponding to an ATP production rate of 25.6 mM h^−1^ (Table 1).

### 5.6. ATP Consumption Kinetics

Two versions of the dependence of the ATP consumption rate on ATP concentration were considered—linear and hyperbolic—described by the following equations:

(29) $$V_{ATPase}^{L}=A_{ATPase}^{L}[ATP]$$

(30) $$V_{ATPase}^{H}=A_{ATPase}^{H}\frac{\left[ATP\right]}{\left[ATP\right]+K_{ATP}}$$

Here, $A_{ATPase}^{L}$, $A_{ATPase}^{H}$, and K_ATP_ are the parameters of ATP-consuming processes, taking the values given in Table 2.

These two versions of the ATP consumption kinetics correspond to cases where the Michaelis constant for the ATP-consuming processes is, respectively, much larger or much smaller than the physiological ATP concentrations.

### 5.7. Steady-State Glycolysis Characteristic

The dependence of the steady-state glycolytic flux on ATP concentration (glycolysis characteristic) was obtained by solving Equations (13) and (14) under steady-state conditions with the change in ATP concentration as a parameter:

(31) $$\frac{d\left[G6P\right]}{dt}={0=V}_{HK}-V_{PGI}$$

(32) $$\frac{d\left[F6P\right]}{dt}=0=V_{PGI}-V_{PFK}$$

In models without AK, AMP concentration was fixed as described above. In models with AK, ADP, and AMP concentrations were calculated from AK equilibrium (Equation (2)) and adenine nucleotide pool conservation (Equation (16)):

(33) $$\left[ADP\right]=\frac{\sqrt{\left[ATP\right]\left(4KA+\left[ATP\right]-4K\left[ATP\right]\right)}-[ATP]}{2K}$$

(34) $$\left[AMP\right]=A-\left[ATP\right]-[ADP]$$

### 5.8. Rate of the Adenylate Kinase Reaction

Our mathematical model includes the AK equilibrium and does not contain an explicit expression for the rate of the AK reaction. However, given that AMP in our model is involved only in the AK reaction, we obtain an expression for the rate of ATP production in the AK reaction as the rate of change in the concentration of AMP:

(35) $$V_{AK}=\frac{d\left[AMP\right]}{dt}$$

Differentiating Equation (24) with respect to time, we obtain the following expression for d[AMP]/dt:

(36) $$\frac{d[AMP]}{dt}=\frac{\left(E-A-Q\right)}{2Q}\cdot \frac{dE}{dt}$$

Here, E = 2[ATP] + [ADP], A = [ATP] + [ADP] + [AMP], and Q is expressed by Equation (25). The expression for dE/dt is given in the model description (Equation (21)). Substituting it into Equation (36) yields the final expression for d[AMP]/dt, which in our model is equal to the rate of ATP production in the AK reaction:

(37) $$\frac{d[AMP]}{dt}=\frac{\left(E-A-Q\right)}{2Q}\cdot \left(-V_{HK}+3V_{PFK}-V_{ATPase}\right)$$

### 5.9. Model Analysis

The kinetics of the models were calculated using the CVODE library [75]. The dependence of the steady states of the models on the parameters was calculated using the KINSOL library [75] and the AUTO 2000 software [76]. The source files of the models can be downloaded from https://doi.org/10.5281/zenodo.18646818 (accessed on 26 February 2026).

## Figures and Tables

**Table 2:** Activities of glycolytic enzymes and parameters of ATP-consuming processes in models of energy metabolism in human erythrocytes and white skeletal muscles of mammals. The range of experimental values taken from [74] is presented in brackets.

Parameter	Human Erythrocytes	Skeletal Muscles	Units
A_HK_—Hexokinase activity	12	100 (27–180)	mM h^−1^
A_GPI_—Glucose phosphate isomerase activity	360	20,000 (15,100–49,800)	mM h^−1^
A_PFK_—Phosphofructokinase activity	380	6000 (2640–11,500)	mM h^−1^
$A_{ATPase}^{L}$—Activity of linear ATPase	1.57	5.16	mM^−1^
$A_{ATPase}^{H}$—Activity of hyperbolic ATPase	2.36	25.7	mM h^−1^
K_ATP_—Michaelis constant of hyperbolic ATPase for ATP	10	10	µM

## Data Availability

The original contributions presented in this study are included in the article/Supplementary Material. Further inquiries can be directed to the corresponding author.

## Supplementary Materials

The following supporting information can be downloaded at: https://www.mdpi.com/article/10.3390/ijms27052479/s1. Reference [77] is cited in the Supplementary Materials.
