# Supplementary material for: The Critical Role of Adenylate Kinase in Regulating the Glycolysis Rate in Cells
Source: Int J Mol Sci. 2026 Mar 8;27(5):2479. doi: 10.3390/ijms27052479 (PMC12985941; doi:10.3390/ijms27052479)
Supplement: Supplementary file 1 [file ijms-27-02479-s001.zip › ijms-4138149-supplementary.pdf]

Supplementary Materials

Dependence of [AMP], [ADP] and [AMP]/[ATP] ratio on [ATP] near the normal physiological steady state of energy metabolism

As noted in the main text, in most cells the normal concentrations of ATP, ADP, and AMP are in a ratio of 100:10:1 (Equation 11). That is, the majority of adenine nucleotides are in the form of ATP. Under these conditions, in the presence of AK, small changes in ATP concentration lead to large changes in AMP concentration. In order to obtain the dependence of the concentrations of AMP and ADP on [ATP] at physiological values of [ATP], we express the concentrations of AMP and ADP in the cell through [ATP], the size of the adenine nucleotide pool, and the equilibrium constant of the AK reaction. Let us write the equations for the pool of adenine nucleotides and for the equilibrium of the adenylate kinase reaction:

$$[ATP] + [ADP] + [AMP] = A \quad \text{(s1)}$$

$$K = \frac{[ATP][AMP]}{[ADP]^2} = 1 \quad \text{(s2)}$$

The solution of this system gives the following equations for the concentrations of ADP and AMP:

$$[ADP] = \frac{\sqrt{[ATP](4AK + [ATP] - 4K[ATP])} - [ATP]}{2K} \quad \text{(s3)}$$

$$[AMP] = A - [ATP] - [ADP] \quad \text{(s4)}$$

From these equations it follows that a decrease in ATP concentration by only 5% from the physiological value leads to a twofold increase in AMP concentration, while ADP concentration increases by only 40%. An increase in ATP concentration by 3% from the physiological value leads to a decrease in AMP concentration by 46%, while the ADP concentration decreases by 25%. Thus, in the presence of AK, the AMP concentration is a very sensitive indicator of the deviation of the ATP concentration from the physiological value in the cell. In order to evaluate the general form of the dependence of the concentrations of AMP and ADP on the concentration of ATP in the normal state of energy metabolism, that is, at [ATP] ≈ A, we will expand the dependence of [AMP] and [ADP] on [ATP] in a series in the vicinity of the physiologically normal value of [ATP]. To do this, we introduce a small parameter δ:

$$[ATP] = A(1 - \delta) \quad \text{(s5)}$$

After expansion in a series with respect to the small parameter δ we obtain:

$$[AMP] = KA\delta^2 + O(\delta^3) \quad \text{(s6)}$$

$$[ADP] = A\delta + O(\delta^2) \quad \text{(s7)}$$

$$\frac{[AMP]}{[ATP]} = K\delta^2 + O(\delta^3) \quad \text{(s8)}$$

Expressing δ through [ATP]

$$\delta = 1 - \frac{[ATP]}{A} \quad \text{(s9)}$$

we obtain the desired dependences of [AMP] and [ADP] on [ATP] in the vicinity of the physiologically normal value of [ATP]:

$$[AMP] \approx AK\left(1 - \frac{[ATP]}{A}\right)^2 \quad \text{(s10)}$$

$$[ADP] \approx A\left(1 - \frac{[ATP]}{A}\right) \quad \text{(s11)}$$

$$\frac{[AMP]}{[ATP]} \approx K\left(1 - \frac{[ATP]}{A}\right)^2 \quad \text{(s12)}$$

<u>Equations for energy metabolism in white skeletal muscle in the presence of creatine-phosphocreatine system and creatine kinase (CK)</u>

In addition to adenylate kinase, skeletal muscle contains the creatine–phosphocreatine system and the enzyme creatine kinase, which buffers ATP concentration in muscle cells via the reaction:

$$CrP + ADP \rightleftarrows Cr + ATP \quad \text{(s13)}$$

Here CrP and Cr are phosphocreatine and creatine respectively.
The equations describing energy metabolism in white skeletal muscle are as follows:

$$\frac{d[ATP]}{dt} = -V_{HK} + 3V_{PFK} - V_{ATPase} + V_{AK} + V_{CK} \quad \text{(s14)}$$

$$\frac{d[ADP]}{dt} = V_{HK} - 3V_{PFK} + V_{ATPase} - 2V_{AK} - V_{CK} \quad \text{(s15)}$$

$$\frac{d[AMP]}{dt} = V_{AK} \quad \text{(s16)}$$

$$\frac{d[Cr]}{dt} = V_{CK} \quad (s17)$$

$$\frac{d[CrP]}{dt} = -V_{CK} \quad (s18)$$

We assume that the activities of adenylate kinase and creatine kinase are high enough to provide the equilibrium ratios between substrates and products of these reactions:

$$K_{AK} = \frac{[ATP][AMP]}{[ADP]^2} \quad (s19)$$

$$K_{CK}[H^+] = K'_{CK} = \frac{[Cr][ATP]}{[CrP][ADP]} \quad (s20)$$

In our calculations, we assume that $[H^+]$ is constant. By taking into account two conservation equations for metabolites involved in energy turnover:

$$[ATP] + [ADP] + [AMP] = A = const \quad (s21)$$

$$[Cr] + [CrP] = C = const \quad (s22)$$

one can express concentrations of ATP, ADP, AMP, and Cr through the concentration of CrP:

$$[Cr] = C - [CrP] \quad (s23)$$

$$[ATP] = \frac{[CrP]^2 K'^2_{CK} A}{D} \quad (s24)$$

$$[ADP] = \frac{(C-[CrP])[CrP]K'_{CK}A}{D} \quad (s25)$$

$$[AMP] = \frac{(C-[CrP])^2 K_{AK} A}{D} \quad (s26)$$

where

$$D = C^2 K_{AK} + C[CrP](K'_{CK} - 2K_{AK}) + [CrP]^2(K_{AK} + (K'_{CK} - 1)K'_{CK}) \quad (s27)$$

Values for C, CrP, and K'_{CK} in the model as well as experimental ranges for these parameters are shown in the Table s1.

From the model kinetic analysis point of view, it is convenient to introduce the energy pool as a new independent variable:

$$E = 2[ATP] + [ADP] + [CrP] \quad (s28)$$

By substituting the expressions (s24, s25) for [ATP] and [ADP] into (s28) and rearranging the resulting equation, one can obtain the cubic equation regarding [CrP] that can be solved to obtain the concentration of CrP as a function of the energy pool (*E*):

$$a[CrP]^3 + b[CrP]^2 + c[CrP] + d = 0 \qquad \text{(s28)}$$

where

$$a = (1 - K'_{CK})K'_{KC} - K_{AK} \qquad \text{(s29)}$$
$$b = C(2K_{AK} - K'_{CK}) + E(K_{AK} + (K'_{CK} - 1)K'_{CK}) - AK'_{CK}(2K'_{CK} - 1) \qquad \text{(s30)}$$
$$c = CE(K'_{CK} - 2K_{AK}) - CK'_{CK}A - C^2K_{AK} \qquad \text{(s31)}$$
$$d = C^2EK_{AK} \qquad \text{(s32)}$$

The equation (s28) is solved using the Cardano formula and taking as a solution a root that is positive at positive *E* values (see the next section).

The differential equation for the kinetics of *E* can be obtained by summing up equations (s14, s15, s18):

$$\frac{dE}{dt} = 2\frac{d[ATP]}{dt} + \frac{d[ADP]}{dt} + \frac{d[CrP]}{dt} = -V_{HK} + 3V_{PFK} - V_{ATPase} \qquad \text{(s33)}$$

The advantage of this equation is the absence of expressions for the fast equilibrium AK and CK reactions.

In its final form, the white muscle mathematical model is a system of three differential equations: (13, 14, s33). Concentrations of energy metabolites were calculated from phase variables using equations (s28, s23 – s26).

Applying the Cardano formula for solving the equation for creatine phosphate concentration.

We used the Cardano formula for solving cubic equation (s28) and for the calculation of [CrP] as a function of *e*:

$$[CrP] = \alpha + \beta - \frac{b}{3a} \qquad \text{(s34)}$$

where

$$\beta = -\frac{p}{3\,\alpha} \qquad \text{(s35)}$$

$$\alpha = \left(-\frac{q}{2} + \theta^{\frac{1}{2}}\right)^{\frac{1}{3}} \quad \text{(s36)}$$

$$\theta = \left(\frac{p}{3}\right)^3 + \left(\frac{q}{2}\right)^2 \quad \text{(s37)}$$

$$q = \frac{2b^3 - 9abc + 27a^2 d}{27a^3} \quad \text{(s38)}$$

$$p = \frac{3ac - b^2}{3a^2} \quad \text{(s39)}$$

The parameter α is a real number at positive values of *e* (energy pool). The parameter p is also a real number. Thus, we calculate β directly from the condition for root selection (s35).

Equation for the rate of AK reaction in the model with CK equilibrium

The expression for the adenylate kinase rate in the white muscle model is obtained by solving the system of algebraic equations (s19–s22) and (s28). By differentiating these equations with respect to time, solving the resulting linear system with respect to the time derivatives allows one to express d[AMP]/dt in terms of dE/dt:

$$V_{AK} = \frac{d[AMP]}{dt} = -\frac{dE}{dt}\frac{([AMP]+2[ADP]K_{AK})([ATP]+[ADP]K'_{CK})}{\mathrm{Z}} \quad \text{(s40)}$$

where

$$Z = [AMP][ATP] + [ATP]^2 + [ATP][Cr] + 4[ADP][ATP]K_{AK} + 2[ADP][Cr]K_{AK} + [ADP][AMP]K'_{CK} \quad \text{(s41)}$$

Table s1. Parameters of the creatine – phosphocreatine system in mammalian white skeletal muscles.

**Parameter**	**Units**	**Model**	**Experiment**	**References**
C = [Cr]+[CrP]	mM	25	9.8 – 28.0	[1–3]
K'_{CK}		5	4.6	[4]
[CrP]	mM	17.8	4.8 – 21	[1–3,5–8]

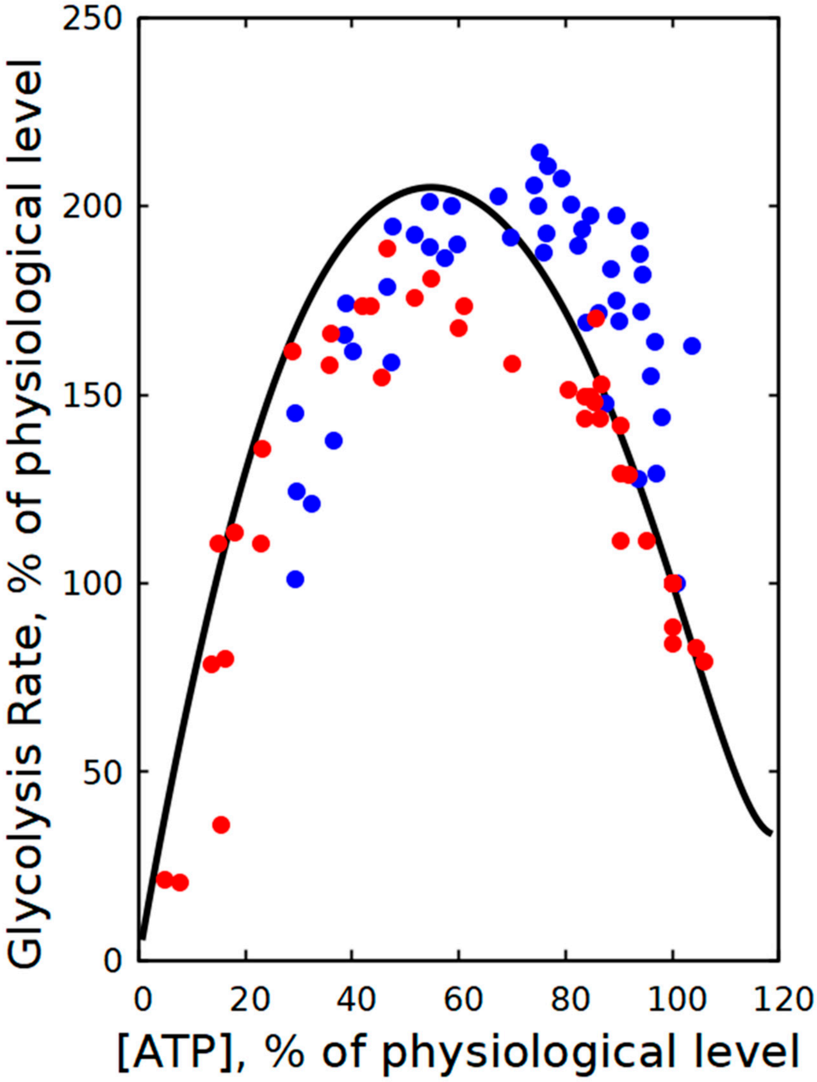

Figure s1. Comparison of theoretical and experimental dependences of the steady-state glycolysis rate on ATP concentration in human erythrocytes, presented in relative coordinates. The solid line shows the steady-state dependence of the glycolysis rate on ATP concentration in the human erythrocyte model obtained in this work using the parameter values specified in the model description. Red and blue symbols represent experimental data obtained in human erythrocytes in studies [9] and [10], respectively (references [42] and [59] in the main text). In each study, erythrocytes from 13 donors were used. A large variability in normal physiological values of glycolysis rate and ATP concentration is observed among erythrocytes from different donors. Therefore, the data are presented in relative coordinates. Normal physiological values of the glycolysis rate and ATP concentration obtained in the model and in each individual experiment with erythrocytes from a given donor were taken as 100%.

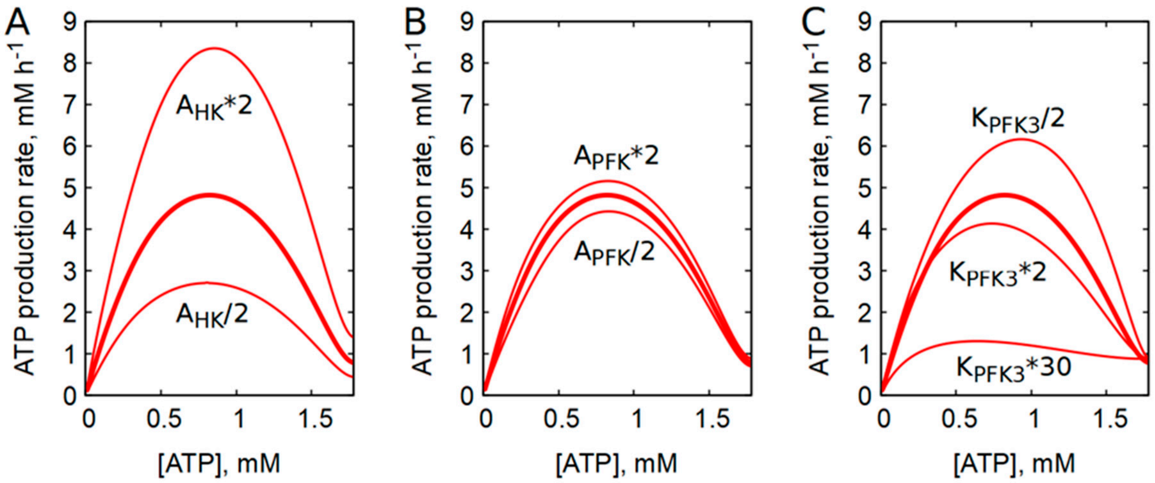

Figure s2. Effect of model parameters in the human erythrocyte model on the steady-state dependence of the rate of ATP production in glycolysis on ATP concentration. (A) effect of hexokinase activity (A_{HK}); (B) effect of phosphofructokinase activity (A_{PFK}); (C) effect of parameter K_{PFK3} in the rate equation for the PFK reaction (equation 28 in the main text), which determines the degree of PFK activation by AMP. Bold curves were obtained using the parameter values specified in the model description. Thin curves were obtained by twofold increase and twofold decrease of the corresponding parameter value (as indicated on the curves). For parameter K_{PFK3}, an additional curve obtained with a 30-fold increase of the parameter value is also shown.

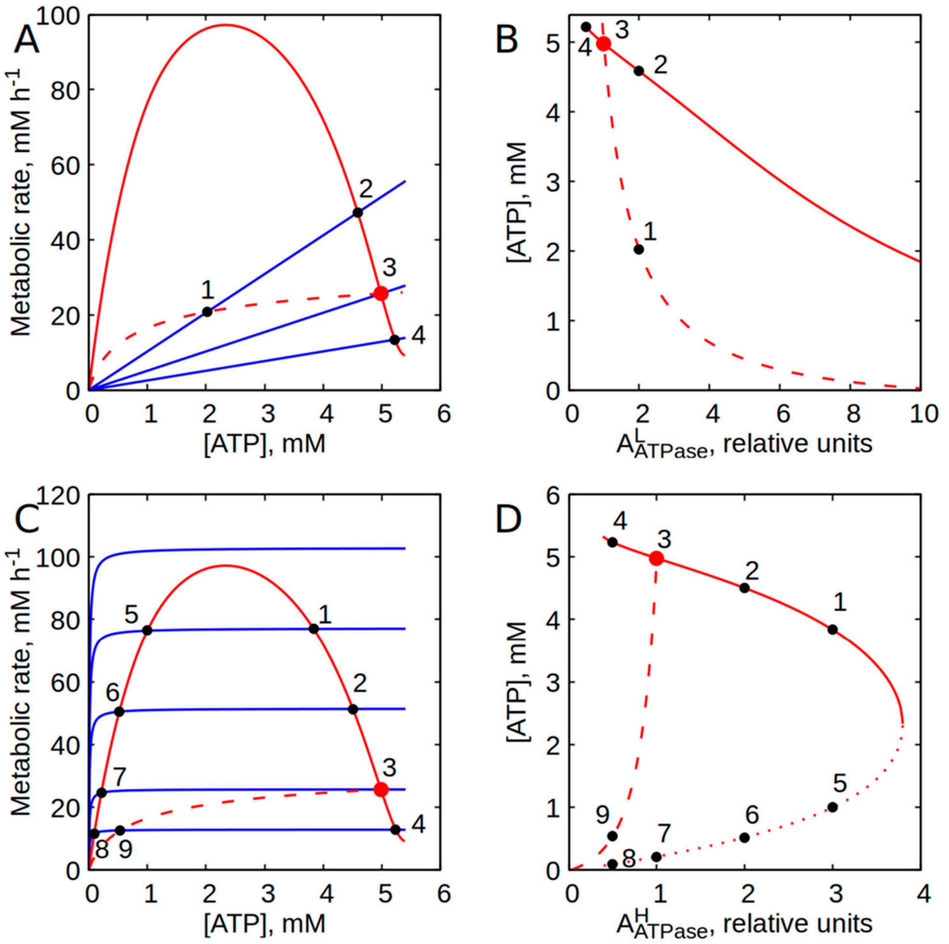

Figure s3. The influence of the AK equilibrium on the regulation of energy metabolism in mammalian white skeletal muscle model. A-Red lines show the steady-state dependences of the rate of ATP production in glycolysis on [ATP] in the presence of AK equilibrium (solid line) and in the absence of AK (dashed line). The blue lines show the dependence of the rate of ATP consumption on [ATP] at three different slope values which are equal to 0.5, 1, and 2 times the normal physiological slope value; B-Stationary dependences of ATP concentration on the activity of ATP-consuming processes, obtained with a linear dependence of the rate of ATP consumption on [ATP] in the presence of AK equilibrium (solid line) and in the absence of AK (dashed line); C-The red lines correspond to the red lines in panel A. The blue lines show the hyperbolic dependence of the rate of ATP consumption on [ATP] at a Michaelis constant of 10 μM and different values of the maximal rate of ATP consumption which are equal to 0.5, 1, 2, 3, and 4 times the normal physiological rate of ATP consumption; D-Stationary dependences of the ATP concentration on the activity of ATP-consuming processes, obtained with a hyperbolic dependence of the rate of ATP consumption on [ATP] with a Michaelis constant value equal to 10 μM in the presence of AK equilibrium (solid and dotted lines) and in the absence of AK (dashed line). In the panel D the dotted and dashed lines correspond to unstable steady states. Black and red circles mark individual stationary states. The same numbers next to the steady states in the left and right panels indicate the same steady states. Red circles indicate physiologically normal steady states. The results were obtained at an adenine nucleotide pool value of 5.4 mM.

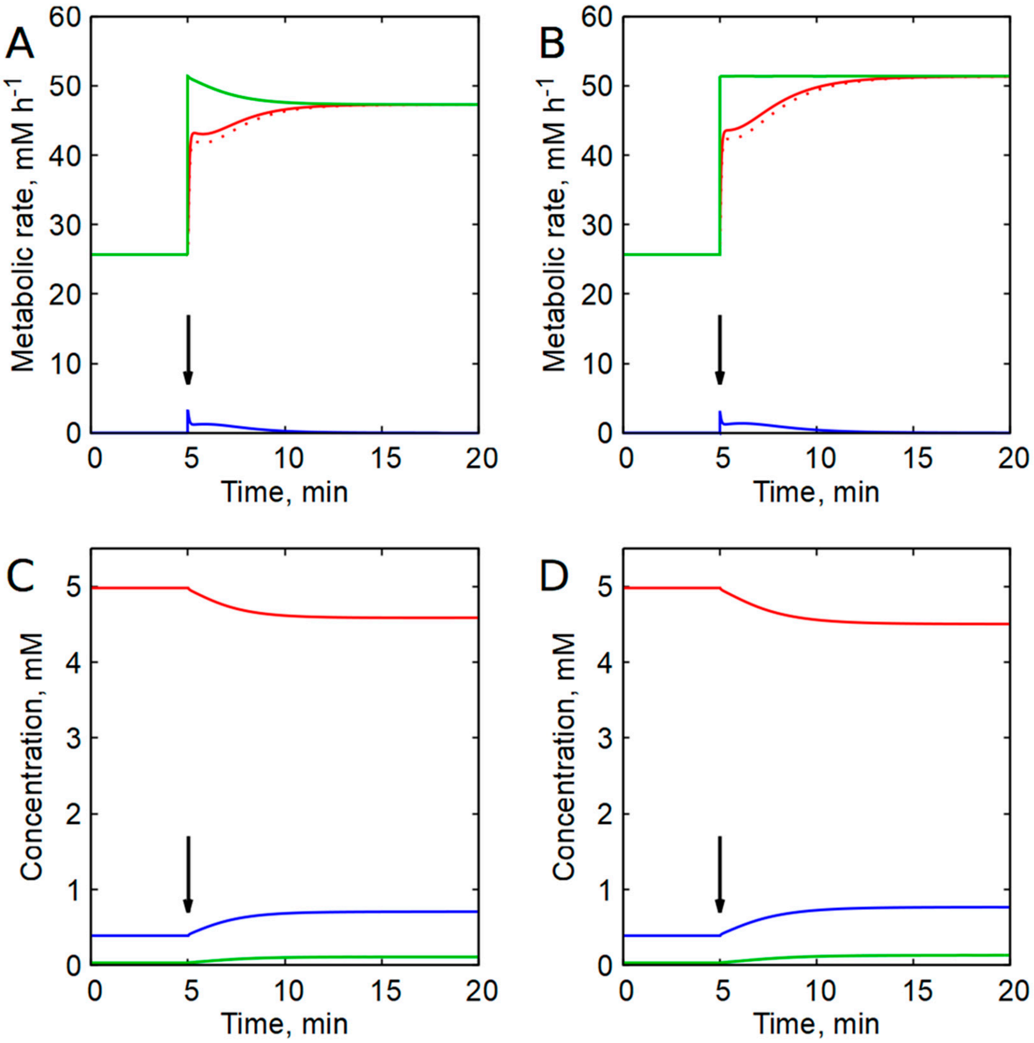

Figure s4. Kinetics of adenine nucleotide levels and the contribution of the AK reaction to the rate of ATP production in mammalian white skeletal muscle model after an instantaneous twofold increase in the activity of ATP-consuming processes in the presence of AK equilibrium but in the absence of creatine kinase (CK). A- Kinetics of the rate of ATP consumption (green line), the total rate of ATP production (red line), the total rate of ATP production minus the AK reaction rate (red dotted line) and the rate of ATP production in the AK reaction (blue line) obtained with a linear dependence of the rate of ATP consumption on [ATP]; B- Kinetics of the rate of ATP consumption (green line), the total rate of ATP production (red line), the total rate of ATP production minus the AK reaction rate (red dotted line) and the rate of ATP production in the AK reaction (blue line) obtained with a hyperbolic dependence of the rate of ATP consumption on [ATP] with a Michaelis constant equal to 10 μM; C- Kinetics of [ATP] (red line), [ADP] (blue line) and [AMP] (green line) obtained with a linear dependence of the rate of ATP consumption on [ATP]; D- Kinetics of [ATP] (red line), [ADP] (blue line) and [AMP] (green line) obtained with a hyperbolic dependence of the rate of ATP consumption on [ATP] with a Michaelis constant equal to 10 μM. The activity of ATP-consuming processes changed at the time indicated by the arrow from 5.16 mM/h to 10.32 mM/h in the case of a linear and from 25.7 mM/h to 51.4 mM/h in the case of a hyperbolic dependence of the rate of ATP consumption on [ATP]. The results were obtained at an adenine nucleotide pool value of 5.4 mM.

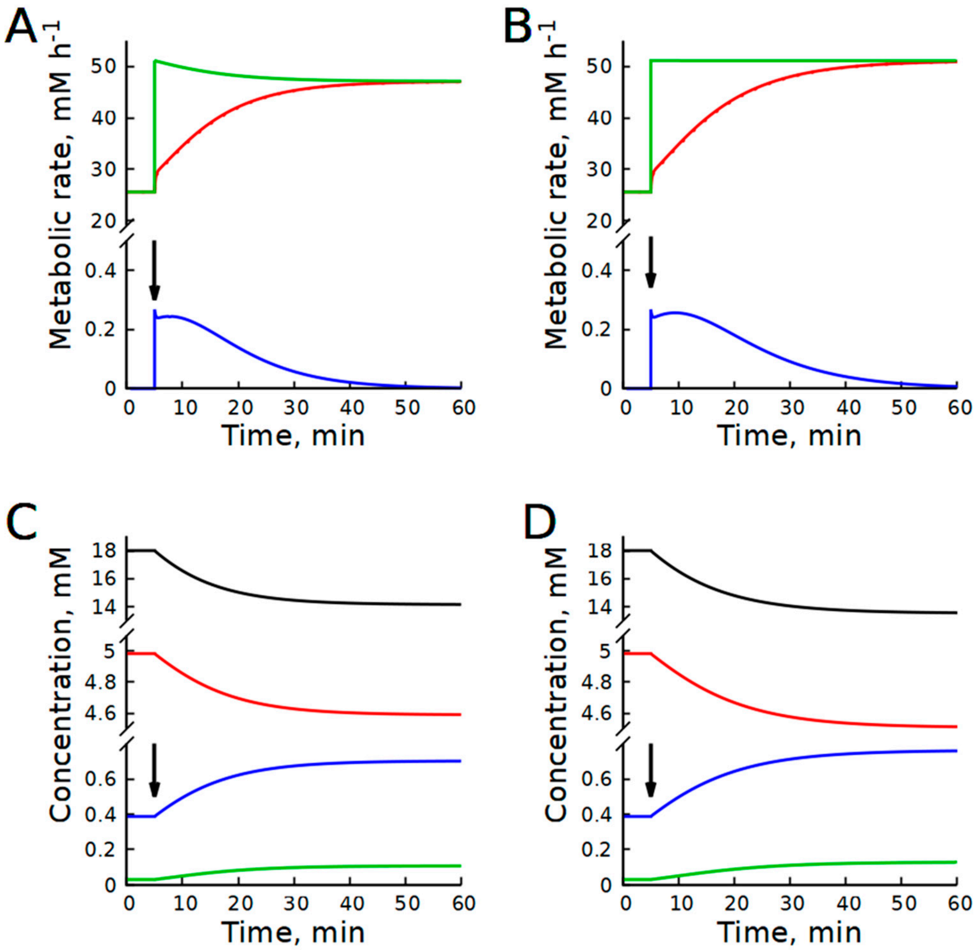

Figure s5. Kinetics of adenine nucleotide levels and the contribution of the AK reaction to the rate of ATP production in mammalian white skeletal muscle model after an instantaneous twofold increase in the activity of ATP-consuming processes in the presence of both, AK equilibrium and creatine kinase (CK) equilibrium. A- Kinetics of the rate of ATP consumption (green line), the total rate of ATP production (red line), and the rate of ATP production in the AK reaction (blue line) obtained with a linear dependence of the rate of ATP consumption on [ATP]; B- Kinetics of the rate of ATP consumption (green line), the total rate of ATP production (red line), and the rate of ATP production in the AK reaction (blue line) obtained with a hyperbolic dependence of the rate of ATP consumption on [ATP] with a Michaelis constant equal to 10 μM; C- Kinetics of [ATP] (red line), [ADP] (blue line), [AMP] (green line), and phosphocreatine concentration (black line) obtained with a linear dependence of the rate of ATP consumption on [ATP]; D- Kinetics of [ATP] (red line), [ADP] (blue line), [AMP] (green line) and phosphocreatine concentration (black line) obtained with a hyperbolic dependence of the rate of ATP consumption on [ATP] with a Michaelis constant equal to 10 μM. The activity of ATP-consuming processes changed at the time indicated by the arrow from 5.16 mM/h to 10.32 mM/h in the case of a linear and from 25.7 mM/h to 51.4 mM/h in the case of a hyperbolic dependence of the rate of ATP consumption on [ATP]. The results were obtained at an adenine nucleotide pool value of 5.4 mM and creatine-phosphocreatine pool value of 25 mM.

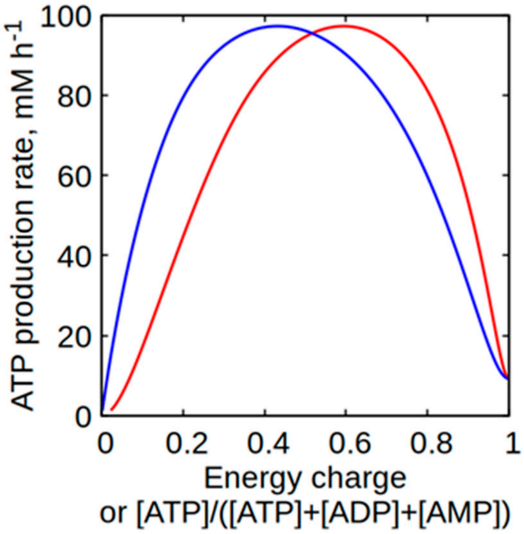

Figure s6. Dependence of the rate of ATP production in glycolysis on the energy charge (red line) and on the relative concentration of ATP ([ATP]/([ATP]+[ADP]+[AMP]) (blue line) in mammalian white skeletal muscle model in the presence of AK equilibrium. The results were obtained with adenine nucleotide pool value equal to 5.4 mM.

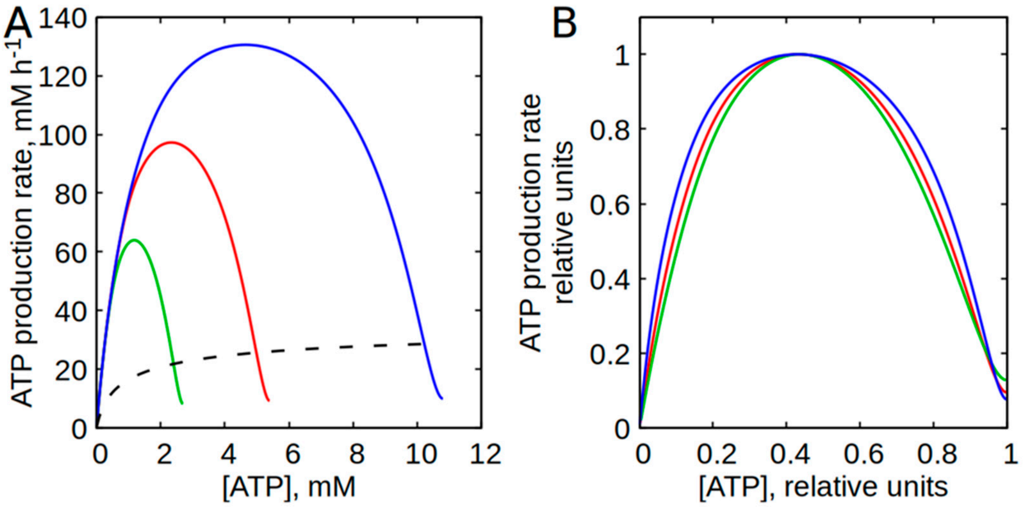

Figure s7. The influence of the AK equilibrium on the steady-state dependence of the glycolysis rate on the ATP concentration in mammalian white skeletal muscle model at different values of the adenine nucleotide pool. A-The solid lines show the steady-state dependences of the rate of ATP production in glycolysis on the absolute concentration of ATP, obtained for adenine nucleotide pool values equal to 10.8 mM (blue line), 5.4 mM (red line) and 2.7 mM (green line) in the presence of AK equilibrium. The dashed line shows the steady-state dependence of the rate of ATP production in glycolysis on the absolute concentration of ATP, obtained in the absence of AK equilibrium at AMP concentration of 30 µM; B-Graphs of the bell-shaped curves shown in panel A, obtained in the presence of AK equilibrium, after normalization to the maximum point.

References

1. Li, Y.; Dash, R.K.; Kim, J.; Saidel, G.M.; Cabrera, M.E. Role of NADH/NAD$^+$ Transport Activity and Glycogen Store on Skeletal Muscle Energy Metabolism during Exercise: In Silico Studies. *Am. J. Physiol.-Cell Physiol.* **2009**, *296*, C25–C46, doi:10.1152/ajpcell.00094.2008.

2. Ren, J.M.; Chasiotis, D.; Bergström, M.; Hultman, E. Skeletal Muscle Glucolysis, Glycogenolysis and Glycogen Phosphorylase during Electrical Stimulation in Man. *Acta Physiol. Scand.* **1988**, *133*, 101–107, doi:10.1111/j.1748-1716.1988.tb08387.x.

3. Harris, R.C.; Hultman, E.; Nordesjö, L.O. Glycogen, Glycolytic Intermediates and High-Energy Phosphates Determined in Biopsy Samples of Musculus Quadriceps Femoris of Man at Rest. Methods and Variance of Values. *Scand. J. Clin. Lab. Invest.* **1974**, *33*, 109–120.

4. Sahlin, K.; Harris, R.C.; Hultman, E. Creatine Kinase Equilibrium and Lactate Content Compared with Muscle pH in Tissue Samples Obtained after Isometric Exercise. *Biochem. J.* **1975**, *152*, 173–180, doi:10.1042/bj1520173.

5. Greenhaff, P.L.; Nevill, M.E.; Soderlund, K.; Bodin, K.; Boobis, L.H.; Williams, C.; Hultman, E. The Metabolic Responses of Human Type I and II Muscle Fibres during Maximal Treadmill Sprinting. *J. Physiol.* **1994**, *478*, 149–155, doi:10.1113/jphysiol.1994.sp020238.

6. Cheetham, M.E.; Boobis, L.H.; Brooks, S.; Williams, C. Human Muscle Metabolism during Sprint Running. *J. Appl. Physiol.* **1986**, *61*, 54–60, doi:10.1152/jappl.1986.61.1.54.

7. Dohm, G.L.; Patel, V.K.; Kasperek, G.J. Regulation of Muscle Pyruvate Metabolism during Exercise. *Biochem. Med. Metab. Biol.* **1986**, *35*, 260–266, doi:10.1016/0885-4505(86)90081-2.

8. Karl, I.E.; Voyles, N.; Recant, L. Effects of Plasma Albumin on Glycolytic Intermediates in Rat Diaphragm Muscle. *Diabetes* **1968**, *17*, 374–384, doi:10.2337/diab.17.6.374.

9. Ataullakhanov, F.I.; Vitvitsky, V.M.; Zhabotinsky, A.M.; Pichugin, A.V.; Platonova, O.V.; Kholodenko, B.N.; Ehrlich, L.I. The Regulation of Glycolysis in Human Erythrocytes: The Dependence of the Glycolytic Flux on the ATP Concentration. *Eur. J. Biochem.* **1981**, *115*, 359–365, doi:10.1111/j.1432-1033.1981.tb05246.x.

10. Platonova, O.V.; Agranenko, V.A.; Ataullakhanov, F.I.; Vitvitskiĭ, V.M.; Kiiatkina, N.V. [Effect of pH on the regulatory characteristics of energy metabolism in human erythrocytes] (in Russian). *Biokhimiia Mosc. Russ.* **1986**, *51*, 1384–1391.
